# Detecting transcriptomic structural variants in heterogeneous contexts via the Multiple Compatible Arrangements Problem

**DOI:** 10.1186/s13015-020-00170-5

**Published:** 2020-05-15

**Authors:** Yutong Qiu, Cong Ma, Han Xie, Carl Kingsford

**Affiliations:** Computational Biology Department, Carnegie Mellon University, 5000 Forbes Ave, 15213 Pittsburgh, PA USA

**Keywords:** Transcriptomic structural variation, Integer linear programming, Heterogeneity

## Abstract

**Background:**

Transcriptomic structural variants (TSVs)—large-scale transcriptome sequence change due to structural variation - are common in cancer. TSV detection from high-throughput sequencing data is a computationally challenging problem. Among all the confounding factors, sample heterogeneity, where each sample contains multiple distinct alleles, poses a critical obstacle to accurate TSV prediction.

**Results:**

To improve TSV detection in heterogeneous RNA-seq samples, we introduce the Multiple Compatible Arrangements Problem (MCAP), which seeks *k* genome arrangements that maximize the number of reads that are concordant with at least one arrangement. This models a heterogeneous or diploid sample. We prove that MCAP is NP-complete and provide a $\frac{1}{4}$-approximation algorithm for $k=1$ and a $\frac{3}{4}$-approximation algorithm for the diploid case ($k=2$) assuming an oracle for $k=1$. Combining these, we obtain a $\frac{3}{16}$-approximation algorithm for MCAP when $k=2$ (without an oracle). We also present an integer linear programming formulation for general *k*. We characterize the conflict structures in the graph that require $k>1$ alleles to satisfy read concordancy and show that such structures are prevalent.

**Conclusions:**

We show that the solution to MCAP accurately addresses sample heterogeneity during TSV detection. Our algorithms have improved performance on TCGA cancer samples and cancer cell line samples compared to a TSV calling tool, SQUID. The software is available at https://github.com/Kingsford-Group/diploidsquid.

## Background

Transcriptomic structural variations (TSVs) are transcriptome sequence alterations due to genomic structural variants (SVs). TSVs may cause the joining of parts from different genes, which are fusion-gene events. Fusion genes are known for their association with various types of cancer. For example, the joint protein products of *BCR*-*ABL1* genes are prevalently found in leukemia [1]. In addition to fusion genes, the joining of intergenic and genic regions, called non-fusion-gene events, are also related to cancer [2].

TSV events are best studied with RNA-seq data. Although SVs are more often studied with whole genome sequencing (WGS) [3–8], the models built on WGS data lack the flexibility to describe alternative splicing and differences in expression levels of transcripts affected by TSVs. In addition, RNA-seq data is far more common [9] than WGS data in some data cohorts, for example, in The Cancer Genome Atlas (TCGA, https://cancergenome.nih.gov).

Many methods have been proposed that identify fusion genes with RNA-seq data. Generally, these tools identify candidates of TSV events through investigation into read alignments that are discordant with the reference genome (e.g. [10–15]). A read alignment is *concordant* with a reference sequence if the alignment to the sequence agrees with the read library preparation. For example in paired-end Illumina sequencing, the orientation of the forward read should be $5'$-to-$3'$ and the reverse for the mate read. Otherwise the alignment is *discordant* with the reference. A series of filtering or scoring functions are applied on each TSV candidate to eliminate the errors in alignment or data preparation. The performance of filters often relies heavily on a large set of method parameters and requires prior annotation [16]. Furthermore, most of the fusion-gene detection methods limit their scope to the joining of protein-coding regions and ignore the joining of intergenic regions that could also affect the transcriptome. An approach that correctly models both fusion-gene and non-fusion-gene events without a large number of ad hoc assumptions is desired.

An intuitive TSV model is the one that describes directly the rearrangement of the genome. For example, when an inversion happens, two double-strand breaks (DSB) are introduced to the genome and the segment between the DSBs is flipped. After a series of SVs are applied to a genome, a rearranged genome is produced. In order to identify the TSVs, we can attempt to infer the rearranged genome from the original genome and keep track of the arrangements of genome segments. Since a model of the complete genome is produced, both fusion-gene and non-fusion-gene events can be detected. A recently published TSV detection tool, SQUID [9], models TSV events in this way by determining a single rearrangement of a reference genome that can explain the maximum number of observed sequencing reads. SQUID finds one arrangement of genome segments such that a maximum number of reads are concordant with it. Novel transcriptomic adjacencies appearing in the arrangement are predicted as TSVs while the ones not appearing are regarded as sequencing or alignment errors.

Despite the generally good performance of SQUID, it relies on the assumption that the sample is homogeneous, i.e. the original genome contains only one allele that can be represented by a single rearranged string. This assumption is unrealistic in diploid (or high ploidy) organisms. When TSV events occur within the same regions on different alleles, read alignments may suggest multiple conflicting ways of placing a segment. Under the homogeneous assumption, conflicting TSV candidates are regarded as errors. Therefore, this assumption leads to discarding the conflicting TSV candidates that would be compatible on separate alleles and therefore limits the discovery of true TSVs. Conflicting SV candidates are addressed in a few SV detection tools such as VariationHunter-CR [6]. However, VariationHunter-CR assumes a diploid genome, and its model is built for WGS data that lacks ability to handle RNA-seq data.

We present an improved model of TSV events in heterogeneous contexts. We address the limitation of the homogeneous assumption by extending the assumption to *k* alleles. We introduce the multiple compatible arrangements problem (MCAP), which seeks, assuming the number of alleles *k* is known, an optimal set of *k* arrangements of segments such that the number of sequencing reads that are concordant with any of the arrangements is maximized. Each arrangement is a permutation and reorientation of all segments from the reference genome, representing the altered sequence of one allele. A connection between segments is predicted as a TSV if its supporting reads are discordant in the original genome but are concordant in any of the *k* arrangements, otherwise the connection either agrees with the reference genome or is considered as errors. We show that MCAP is NP-complete. To address NP-completeness, we propose a $\frac{1}{4}$-approximation algorithm for the $k=1$ case and a $\frac{3}{4}$-approximation solution to the $k=2$ case using an oracle for $k=1$. Combining these, we obtain a $\frac{3}{16}$-approximation algorithm for MCAP when $k=2$ (without an oracle). We also present an integer linear programming (ILP) formulation that gives an optimal solution for general *k*.

We characterize the patterns of reads that result in conflicting TSV candidates under a single-allele assumption. We show that these patterns are prevalent in both cancer cell lines and TCGA samples, thereby further motivating the importance of SV detection approaches that directly model heterogeneity.

We apply our algorithms to 381 TCGA samples from 4 cancer types and show that many more TSVs can be identified under a diploid assumption compared to a haploid assumption. We also evaluate an exact ILP formulation under a diploid assumption (D-SQUID) on previously annotated cancer cell lines HCC1395 and HCC1954, identifying several previously known and novel TSVs. We also show that, in most of the TCGA samples, the performance of the approximation algorithm is very close to optimal and the worst case of $\frac{3}{16}$-approximation is rare.

## The Genome Segment Graph (GSG)

A Genome Segment Graph, similar to a splice graph [17], encodes relationships between genomic segments and a set of reads. A *segmentationS* of the genome is a partition of the genome into disjoint intervals according to concordant and discordant paired-end alignments with respect to the reference genome. The genome partitioning, edge construction and edge filtering is done in the same way as in Ma et al. [9].

### **Definition 1**

(*Genome Segment Graph*) A *genome segment graph* is a weighted, undirected graph $G = (V, E, {\mathbf {w}})$ derived from a segmentation *S* of the genome and a collection of reads. The vertex set, $V = \{s_h \in S\}\bigcup \{s_t \in S\}$, includes a vertex for both endpoints, head (*h*) and tail (*t*), for each segment $s\in S$. The head of a segment is the end that is closer to the $5'$ end of the genome. The tail is the end that is close to the $3'$ end. Pairs of reads that span more than one segment are represented by edges. There are four types of connections: head-head, head-tail, tail-head and tail-tail. Each edge $e = (u_i, v_j)\in E$, where $i,j \in \{h, t\}$, is undirected and connects endpoints of two segments. The weight ($w_e \in {\mathbf {w}}$) is the number of sequencing reads that support edge *e*.

We also define the weight of a subset $E' \subseteq E$ of edges $w(E') = \sum _{e\in E'} w_e$. (More details on the GSG provided in Ma et al. [9]).

### **Definition 2**

(*Permutation*, *orientation function and arrangement*) A permutation is a function where $\pi (u) = i$, where *i* is the index of segment $u \in S$ in an ordering of a set *S* of segments. We also define orientation function $f(u) = 1$ if segment *u* should remain in the original orientation, or 0 if it should be inverted. An *arrangement* is a pair of permutation and orientation functions $(\pi , f)$.

If $\pi (u) <\pi (v)$, we say that segment *u* is closer to the $5'$ end of the rearranged genome than segment *v*. Each arrangement is a concatenation of segments from different chromosomes, which retrieves the sequences affected by inter- and intra-chromosomal TSV events. The arrangement of genome segments imitates the movements of genomic sequences by SVs. One crucial difference between arrangement in GSG and sequence movements by SVs is that an arrangement in GSG only captures the movement that are relevant to transcriptome sequence alterations. Such alterations can either fuse two transcript sequences or incorporate previously non-transcribing sequences into transcripts as long as they are present in RNA-seq reads.

### **Definition 3**

(*Concordant and discordant edges*) Let *e* be an edge connecting segment *u* on end *a* and segment *v* on end *b* ($a,b \in \{h,t\}$). Given arrangement $(\pi , f)$, suppose $\pi (u) < \pi (v)$, edge *e* is *concordant* with respect to the arrangement if $f(u) = {\mathbf {1}}[a=t]$ and $f(v)={\mathbf {1}}[b=h]$. Denote the concordance as $e\sim (\pi , f)$. Otherwise, *e* is *discordant* and denote as $e\not \sim (\pi , f)$.

Combining the permutation and orientation function, the edge concordance condition can be equivalently expressed as

$$\begin{aligned} f(u){\mathbf {1}}[a=t] + (1-f(u)){\mathbf {1}}[a=h] = {\mathbf {1}}[\pi (u) < \pi (v)] = f(v){\mathbf {1}}[b=h] + (1-f(v)){\mathbf {1}}[b=t] \end{aligned}$$

**Fig. 1:**
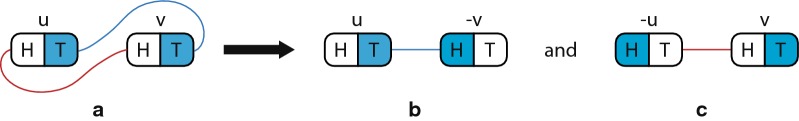
MCAP resolves conflicts. The white ends of the segments represent head with respect to the original genome. The blue ends represent tail with respect to the original genome. “H” stands for head and “T” stands for tail in the current arrangement. **a** Two conflicting edges connecting two segments *u* and *v*. If the sample is known to be homogeneous ($k=1$), then the conflict is due to errors. If $k=2$, MCAP seeks to separate two edges into two compatible arrangements as in **b** and **c**. **b** In the first arrangement, segment *v* is flipped, which makes the blue edge concordant. **c** In the second arrangement, *u* is flipped to make the red edge concordant

Since edges are constructed based on segment connections indicated by read alignments, the concordance and discordance of edges are extensions from read alignments. A discordant edge represents a set of discordant read alignments. Examples of discordant edges with tail-tail and head-head connections are shown in Fig. 1a. Concordant edges, when connecting nodes that belong to the same chromosome, represent concordant alignments that are either continuous alignments or split-alignments due to alternative splicing. Due to alternative splicing, a node can be incident to multiple concordant edges given an arrangement. Edges that initially spanned two chromosomes but become concordant in an arrangement represent inter-chromosomal translocation events.

Segments connected by discordant edges can be arranged so that some of the discordant edges become concordant. See Fig. 1b,c for examples of arrangements that make tail-tail and head-head connections concordant.

### **Definition 4**

(*Conflicts among a set of edges*) Given GSG $G = (V, E, {\mathbf {w}})$ and a subset of edges $E'$, the edges in set $E'$ are in conflict with each other if there is no single arrangement $(\pi , f)$ such that $e \sim (\pi , f)\;(\forall e \in E')$. Otherwise, edges in set $E'$ are compatible with each other.

### **Definition 5**

(*Transcriptomic structural variant(TSV)*) A TSV is a new adjacency in transcript sequences that cannot be explained by alternative splicing.

In GSG, the adjacencies in transcript sequences are represented by edges. New adjacencies that cannot be explained by alternative splicing belong to one of two categories: (1) the set of edges discordant with respect to the original arrangement but concordant in the rearranged genome, (2) edges concordant in both the original and the rearranged genomes that connect segments that are further apart than a user-specified distance, or from different chromosomes. Edges in both categorites are ouput as TSVs. Here, as in Ma et al. [9], edges in the second category are identified during a post-processing step in the implementation.

## The Multiple Compatible Arrangements Problem (MCAP)

### Problem statement

Given an input GSG $G = (V,E,w)$ and a positive integer *k*, the multiple compatible arrangements problem seeks a set of *k* arrangements $A = \{(\pi _i, f_i)\}_{i=1}^k$ that are able to generate the maximum number of sequencing reads:

1 $$\begin{aligned} \max _{A}\, \sum _{e \in E}w(e)\cdot {\mathbf {1}}\left[ e\sim A\right] , \end{aligned}$$

where ${\mathbf {1}}\left[ e\sim A\right]$ is 1 if edge *e* is concordant in at least one $(\pi _i, f_i) \in A$, and 0 otherwise.

This objective function aims to find an optimal set of *k* arrangements of segments where the sum of concordant edge weights is maximized in the arranged alleles, where *k* is the number of alleles and assumed to be known. The objective seeks to maximize the agreement between arranged allelic sequences and observed RNA-seq data. Assuming that the majority of RNA-seq reads are sequenced correctly, the concordant edges with respect to the optimal set of arrangements represent the most confident transcriptomic adjacencies. In heterogeneous samples where $k\ne 1$, MCAP separates the conflicting edges onto *k* alleles as shown in an example in Fig. 1.

When $k=1$, the problem reduces to finding a single arranged genome to maximize the number of concordant reads, which is the problem that SQUID [9] solves. We refer to the special case when $k=1$ as single compatible arrangement problem (SCAP).

Predicted TSVs are the concordant edges with respect to any of the arrangements in a solution to MCAP that were either discordant with respect to the reference genome or spanning multiple chromosomes.

## NP-completeness of SCAP and MCAP

### **Theorem 1**

*SCAP is NP-complete.*

### *Proof*

We prove the NP-completeness by reducing from the Fragment Orientation Problem (FOP) that has been formulated and studied by Kececioglu et al. [18]. In FOP, for any pair of fragments, there is evidence supporting or against that they have the same orientation. FOP maximizes the agreement with the evidence by assigning the fragment orientation. We rephrase the problem statement as follows.

Input: A set of fragments ${\mathcal {F}}$ and a score function $S: {\mathcal {F}} \times \{0,1\} \times {\mathcal {F}} \times \{0,1\} \rightarrow {\mathbb {R}}_+$ that satisfies the following two conditions:

$$\begin{aligned}&S(F_i, o_i, F_j, o_j) = S(F_j, o_j, F_i, o_i) \\&S(F_i, o_i, F_j, o_j) = S(F_i, 1 - o_i, F_j, 1 - o_j) \end{aligned}$$

Output: An orientation of fragments $O: {\mathcal {F}} \rightarrow \{0,1\}$.

Objective: Maximize the sum of score according to the orientation,

$$\begin{aligned} \max _{O} \sum _{F_i, F_j \in {\mathcal {F}}, F_i \ne F_j} S(F_i, O(F_i), F_j, O(F_j)). \end{aligned}$$

Kececioglu et al. [18] defined two symmetric functions and used them to express the objective function in a more specific way:

$$\begin{aligned} \max _{O} \sum _{F_i, F_j \in {\mathcal {F}}, F_i \ne F_j} same(F_i, F_j){\mathbf {1}}[O(F_i) = O(F_j)] + opp(F_i, F_j){\mathbf {1}}[O(F_i) \ne O(F_j)], \end{aligned}$$

where $same: {\mathcal {F}} \times {\mathcal {F}} \rightarrow {\mathbb {R}}_+$ is defined as $same(F_i, F_j) \triangleq S(F_i, 0, F_j, 0) = S(F_i, 1, F_j, 1)$, and $opp: {\mathcal {F}} \times {\mathcal {F}} \rightarrow {\mathbb {R}}_+$ is defined as $opp(F_i, F_j) \triangleq S(F_i, 0, F_j, 1) = S(F_i, 1, F_j, 0)$.

Given any FOP instance, a SCAP instance is constructed in polynomial time by constructing a segment for each fragment in ${\mathcal {F}}$ and assigning edge weights based on the *same* and *opp* function values. Specifically, for fragment $F_i$, construct a segment $s^i$. For any pair of segments $(s^i, s^j)$ construct four edges with the following weights: $w(e=(s^i_h, s^j_h)) = opp(F_i, F_j)$, $w(e=(s^i_t, s^j_h)) = same(F_i,F_j)$, $w(e=(s^i_h, s^j_t)) = same(F_i, F_j)$, and $w(e=(s^i_t, s^j_t)) = opp(F_i, F_j)$. Due to the correspondence between segments *S* and fragments ${\mathcal {F}}$, they can be viewed as parameter substitution and used in interchangeably in FOP and SCAP.

Because the constructed GSG is a complete graph except that there is no within-segment edges, the maximization of SCAP over permutation $\pi$ and orientation *f* can be rewritten as

$$\begin{aligned}&\max _{\pi , f} \sum _{e} w(e) {\mathbf {1}}[e \sim (\pi , f)] \\&\quad = \max _{\pi , f} \sum _{s^i, s^j \in S, s^i \ne s^j} w(e=(s^i_h, s^j_h)){\mathbf {1}}[(s^i_h, s^j_h) \sim (\pi , f)] + w(e=(s^i_t, s^j_h)){\mathbf {1}}[(s^i_t, s^j_h) \sim (\pi , f)] \\&\quad +w(e=(s^i_h, s^j_t)){\mathbf {1}}[(s^i_h, s^j_t) \sim (\pi , f)] + w(e=(s^i_t, s^j_t)){\mathbf {1}}[(s^i_t, s^j_t) \sim (\pi , f)] \\&\quad = \max _{\pi , f} \sum _{s^i, s^j \in S, s^i \ne s^j} opp(s^i, s^j){\mathbf {1}}\left\{ 1-f(s^i) = {\mathbf {1}}[\pi (s^i)< \pi (s^j)] = f(s^j)\right\} \\&\quad +same(s^i,s^j){\mathbf {1}}\left\{ f(s^i) = {\mathbf {1}}[\pi (s^i)< \pi (s^j)] = f(s^j)\right\} \\&\quad +same(s^i,s^j){\mathbf {1}}\left\{ 1 - f(s^i) = {\mathbf {1}}[\pi (s^i)< \pi (s^j)] = 1 - f(s^j)\right\} + \\&\quad opp(s^i, s^j){\mathbf {1}}\left\{ f(s^i) = {\mathbf {1}}[\pi (s^i) < \pi (s^j)] = 1 - f(s^j)\right\} \\&\quad = \max _{\pi , f} \sum _{s^i, s^j \in S, s^i \ne s^j} opp(s^i, s^j){\mathbf {1}}[1-f(s^i) = f(s^j)] + same(s^i,s^j){\mathbf {1}}[f(s^i) = f(s^j)] \\&\quad = \max _{f} \sum _{s^i, s^j \in S, s^i \ne s^j} opp(s^i, s^j){\mathbf {1}}[1-f(s^i) = f(s^j)] + same(s^i, s^j){\mathbf {1}}[f(s^i) = f(s^j)] \end{aligned}$$

In the last step of the above equation, since the objective function does not contain permutation $\pi$, we can take $\pi$ out of the optimization parameter. That means for any permutation the maximum sum of concordant edge weights is the same. Applying reparameterization by changing segment $s^*$ to fragment $F_*$ and changing the segment orientation function *f* with fragment orientation function *O*, the above maximization problem is the same as FOP. As a result, the optimal solution of SCAP and FOP can be used interchangeably to maximize the criterion of each other.

Therefore, given any instance of FOP, an instance of SCAP can be constructed in polynomial time whose solution contains an orientation function that maximized FOP instance at the same time. Since FOP is NP-complete, SCAP is also NP-complete. $\square$

### **Corollary 1**

*MCAP is NP-complete.*

### *Proof*

SCAP is a special case of MCAP with $k=1$, so the NP-completeness of MCAP is immediate. $\square$

## A $\frac{1}{4}$-approximation algorithm for SCAP

We provide a greedy algorithm for SCAP that achieves at least $\frac{1}{4}$ approximation ratio and takes *O*(|*V*||*E*|) time. The main idea of the greedy algorithm is to place each segment into the current order one by one by choosing the current “best” position. The current “best” position is determined by the concordant edge weights between the segment to be placed and the segments already in the current order. 
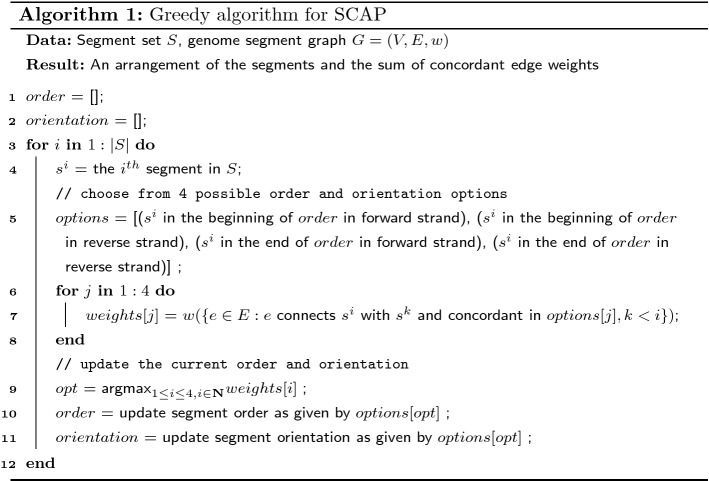


### **Theorem 2**

*Algorithm 1 approximates SCAP with at least* $\frac{1}{4}$*approximation ratio.*

### *Proof*

Denote $E' \subset E$ as the concordant edges in the arrangement of Algorithm 1. Let *OPT* be the optimal value of SCAP. We are to prove $w(E') \ge \frac{1}{4} w(E) \ge \frac{1}{4}OPT$.

For iteration *i* in the for loop, the edges $E_i = \{e\in E: e \text { connects }s^i\text { with }s^j, j < i\}$ are considered when comparing the options. Each of the four options makes a subset of $E_i$ concordant. These subsets are non-overlapping and their union is $E_i$. Specifically, the concordant edge subset is $\{e = (s^i_h, s^j_t): j < i\}$ for the first option, $\{e = (s^i_h, s^j_h): j < i\}$ for the second, $\{e = (s^i_t, s^j_h): j < i\}$ for the third, and $\{e = (s^i_t, s^j_t): j < i\}$ for the last.

By the selecting the option with the largest sum of concordant edge weights, the concordant edges $E'_i$ in iteration *i* satisfies $w(E'_i) \ge \frac{1}{4} w(E_i)$. Therefore, the overall concordant edge weights of all iterations in the for loop satisfy

$$\begin{aligned} \sum _i w(E'_i) \ge \frac{1}{4} \sum _i w(E_i) = \frac{1}{4} w\left( \bigcup _i E_i\right) \,. \end{aligned}$$

Each edge $e\in E$ must appear in one and only one of $E_i$, and thus $\bigcup _i E_i = E$. This implies $\sum _i w(E'_i) \ge \frac{1}{4} w(E) \ge \frac{1}{4} OPT$. $\square$

Algorithm 1 can be further improved in practice by considering more order and orientation options when inserting a segment into current order. In Algorithm 1, only two possible insertion places are considered: the beginning and the end of the current order. However, a new segment can be inserted between any pair of adjacent segments in the current order. We provide an extended greedy algorithm to take into account the extra possible inserting positions (Algorithm 2). Algorithm 2 has a time complexity of $O(|V|^2|E|)$, but it may achieve a higher total concordant edge weight in practice. 
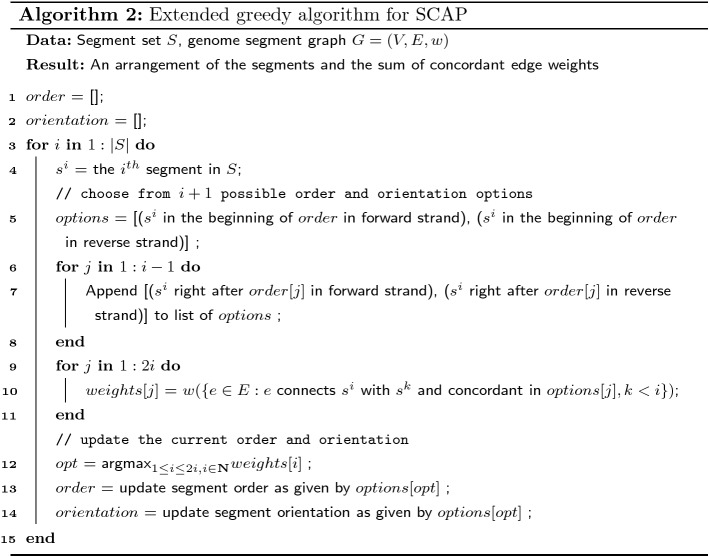


## A $\frac{3}{4}$-approximation of MCAP with $k=2$ using a SCAP Oracle

If an optimal SCAP solution can be computed, one way to approximate the MCAP’s optimal solution is to solve a series of SCAP instances iteratively to obtain multiple arrangements. Here, we prove the solution based on iteratively solving SCAP has an approximation ratio of $\frac{3}{4}$ for the special case of MCAP with $k=2$. 
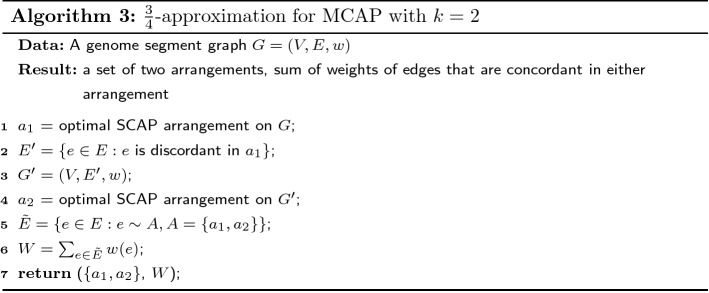


### **Theorem 3**

*Algorithm 3 is a*$\frac{3}{4}$-*approximation of MCAP with*$k=2$. *Denote the optimal objective sum of edge weights in MCAP with*$k=2$*asOPT*, *and the sum of edge weights in the two iterative SCAP asW*, *then*

$$\begin{aligned} W \ge \frac{3}{4} OPT \end{aligned}$$

### *Proof*

Denote MCAP with $k=2$ as 2-MCAP. Let $E_1^d$ and $E_2^d$ be concordant edges in the optimal two arrangements of 2-MCAP. It is always possible to make the concordant edges of the arrangements disjoint by removing the intersection from one of the concordant edge set, that is $E_1^d \cap E_2^d = \emptyset$. Let $E^d = E_1^d \cup E_2^d$. The optimal value is $w(E^d)$.

Denote the optimal set of concordant edges in the first round of Algorithm 3 as $E_1^s$. The optimal value of SCAP is $w(E_1^s)$. $E_1^s$ can have overlap with the two concordant edge sets of the 2-MCAP optimal solution. Let the intersections be $I_1 = E_1^d \cap E_1^s$ and $I_2 = E_2^d \cap E_1^s$. Let the unique concordant edges be $D_1 = E_1^d - E_1^s$, $D_2 = E_2^d - E_1^s$ and $S = E_1^s - E_1^d - E_2^d$.

After separating the concordant edges in 2-MCAP into the intersections and unique sets, the optimal value of 2-MCAP can be written as $w(E^d) = w(I_1) + w(I_2) + w(D_1) + w(D_2)$, where the four subsets are disjoint. Therefore the smallest weight among the four subsets must be no greater than $\frac{1}{4} w(E^d)$. We prove the approximation ratio under the following two cases and discuss the weight of the second round of SCAP separately:

*Case (1): the weight of either*$D_1$*or*$D_2$*is smaller than*$\frac{1}{4}w(E^d)$. Because the two arrangements in 2-MCAP are interchangeable, we only prove for the case where $w(D_1) \le \frac{1}{4} w(E^d)$. A valid arrangement of the second round of SCAP is the second arrangement in 2-MCAP, though it may not be optimal. The maximum concordant edge weights added by the second round of SCAP must be no smaller than $w(D_2)$. Combining the optimal values of two rounds of SCAP, the concordant edge weight is

2 $$\begin{aligned} \begin{aligned} W&\ge w(E_1^s) + w(D_2) = w(S) + w(I_1) + w(I_2) + w(D_2) \\&\ge w(E^d) - w(D_1) \\&\ge \frac{3}{4} w(E^d). \end{aligned} \end{aligned}$$

*Case (2): both*$w(D_1) \ge \frac{1}{4} w(E^d)$*and*$w(D_2) \ge \frac{1}{4} w(E^d)$. The subset with smallest sum of edge weights is now either $I_1$ or $I_2$. Without loss of generality, we assume $I_1$ has the smallest sum of edge weights and $w(I_1) \le \frac{1}{4} w(E^d)$. Because the first round SCAP is optimal for the SCAP problem, its objective value should be no smaller than the concordant edge weights of either arrangement in 2-MCAP. Thus

3 $$\begin{aligned} w(E_1^s) \ge w(E_2^d) = w(D_2) + w(I_2). \end{aligned}$$

A valid arrangement for the second round of SCAP can be either of the arrangements in 2-MCAP optimal solution. Picking the first arrangement of 2-MCAP as the possible (but not necessarily optimal) arrangement for the second round of SCAP, the concordant edge weights added by the second round of SCAP must be no smaller than $w(D_1)$. Therefore, the total sum of concordant edge weights of the optimal solutions of both rounds of SCAP is

4 $$\begin{aligned} \begin{aligned} W&\ge w(E_1^s) + w(D_1) \\&\ge w(D_2) + w(I_2) + w(D_1) \\&= w(E^d) - w(I_1) \\&\ge \frac{3}{4} w(E^d). \end{aligned} \end{aligned}$$

$$\square$$

### **Corollary 2**

*An approximation algorithm for MCAP with*$k=2$*can be created by using Algorithm 1 as the oracle for SCAP in Algorithm 3. This approximation algorithm runs inO*(|*V*||*E*|) *time and achieves at least*$\frac{3}{16}$*approximation ratio.*

The proof of the corollary is similar to the proof of Theorem 3. By adding a multiplier of $\frac{1}{4}$ to the right of inequalities (3) when lower bounding $w(E_1^s)$ by $w(E_2^d)$, the $\frac{3}{16}$ approximation ratio can be derived accordingly.

## Integer linear programming formulation for MCAP

MCAP, for general *k*, can be formulated as an integer linear programming (ILP) to obtain an optimal solution. We rewrite the *i*-th permutation ($\pi _i$), orientation ($f_i$) and decision (${\mathbf {1}}[e\sim (\pi _i, f_i)]$) functions with three boolean variables $y_e^i$, $z_e^i$ and $x_e^i$. For $i \in \{1,2,\ldots ,k\}$ and $e\in E$, we have:

- $x^i_e = 1$ if edge $e\sim (\pi _i,f_i)$ and 0 otherwise.
- $y^i_u = 1$ if $f_i(u) = 1$ for segment *u* and 0 if $f_i(u) = 0$.
- $z^i_{uv} = 1$ if $\pi _i(u) < \pi _i(v)$, or segment *u* is in front of *v* in arrangement *i* and 0 otherwise.

In order to account for the edges that are concordant in more than one arrangement in the summation in Equation 1, we define $q_e$ such that $q_e = 1$ if edge *e* is concordant in one of the *k* arrangements and 0 otherwise. The constraints for $q_e$ are as follows:

5 $$\begin{aligned} q_e&\le \sum _i^k x^i_e \end{aligned}$$

6 $$\begin{aligned} q_e&\le 1 \end{aligned}$$

The objective function becomes

7 $$\begin{aligned} \max _{x^i_e, y^i_u, z^i_{uv}} \,\sum _{e\in E} w(e)\cdot q_e \end{aligned}$$

We then add ordering and orientation constraints. If an edge is a tail-head connection, i.e. concordant to the reference genome, $x^i_e = 1$ if and only if $z^i_{uv} = y^i_u = y^i_v$. If an edge is a tail-tail connection, $x^i_e=1$ if and only if $z^i_{uv} = 1-y^i_v = y^i_u$. If an edge is a head-tail connection, $x^i_e = 1$ if and only if $z^i_{uv} = 1-y^i_u = 1-y^i_v$. If an edge is a head-head connection, $x^i_e =1$ if and only if $z^i_{uv} = 1-y^i_u = y^i_v$. The constraints for a tail-head connection are listed below in Equation 8, which enforce the assignment of boolean variables $y_e^i$, $z_e^i$ and $x_e^i$:

8 $$\begin{aligned} \begin{aligned} x^i_e&\le y^i_u - y^i_v + 1,\\ x^i_e&\le y^i_v - y^i_u + 1,\\ x^i_e&\le y^i_u - z^i_{uv} + 1,\\ x^i_e&\le z^i_{uv} - y^i_u + 1,\\ \end{aligned} \end{aligned}$$

The constraints of other types of connections are similar and detailed in Ma et al. [9]. Additionally, constraints are added so that all segments are put into a total order within each allele. For two segments *u*, *v*, segment *u* will be either precede or follow segment *v*, i.e. $z^i_{uv} + z^i_{vu} = 1$. For three segments *u*, *v*, *w*, if *u* precedes *v* and *v* precedes *w*, then *u* has to precede *w*: $1 \le z^i_{uv}+z^i_{vw}+z^i_{wu} \le 2$.

The total number of constraints as a function of *k* is $$4k|E| + k\left( {\begin{array}{c}|V|\\ 3\end{array}}\right) + 2|E| = O(k(|E|+V^3))$$. When *k* increases, the number of constraints grows linearly. When $k=1$, the ILP formulation reduces to the same formulation as SQUID.

## Characterizing the conflict structures that imply heterogeneity

In this section, we ignore edge weights and characterize the graph structures where homogeneous assumption cannot explain all edges. We add a set of segment edges, ${\hat{E}}$, to the GSG. Each ${\hat{e}}\in {\hat{E}}$ connects the two endpoints of each segment, i.e. ${\hat{e}} = \{s_h, s_t\}$ for $s \in S$. The representation of GSG becomes $G = (E, {\hat{E}}, V)$.

### **Definition 6**

(*Conflict structures and compatible structures*) A *conflict structure*, $CS = (E', {\hat{E}}', V')$, is a subgraph of a GSG where there exists a set of edges $E'$ that cannot be made concordant using any single arrangement. A *compatible structure* is a subgraph of a GSG where there exists a single arrangement such that all edges can be made concordant in it.

### **Definition 7**

(*Simple cycle in GSG*) A *simple cycle*, $C = (E',{\hat{E}}', \{v_0,\dots ,v_{n-1}\})$, is a subgraph of a GSG, such that $E' \subseteq E, {\hat{E}}' \subseteq {\hat{E}}$ and $v_i \in V$, with $(v_i, v_{(i+1) \mod n}) \in E'\cup {\hat{E}}'$ and where $v_i \ne v_j$ when $i\ne j$ except $v_{n-1} = v_0$.

### **Definition 8**

(*Degree and special degree of a vertex in subgraphs of GSG*) Given a subgraph of GSG, $G' = (E', \hat{E'}, V')$, $deg_{E'}(v)$ refers to the degree of vertex $v\in V'$ that counts only the edges $e\in E'$ that connect to *v*. *deg*(*v*) refers to the number of edges $e\in E'\cup \hat{E'}$ that connect to *v*.

### **Theorem 4**

*Any acyclic subgraph of GSG is a compatible structure.*

### *Proof*

We show that any acyclic subgraph with *N* edges ($|E'| + |{\hat{E}}'| = N$), $G'_N = (E', {\hat{E}}', V')$, of GSG is a compatible structure by induction.

When $|E'| + |{\hat{E}}'| = 1$, $G'_1$ is a compatible structure because no other edge in $G'$ is in conflict with the only edge $e\in E'$.

Assume the theorem hold for any acyclic subgraph that contains *n* edges. Let $G'_{n+1} = (E', {\hat{E}}, V')$ be an acyclic subgraph with $n+1$ edges. Since $G'_{n+1}$ is acyclic, there must be a leaf edge that is incident to a leaf node. Denote the leaf node as $v_b$ and the leaf edge $e=(u_a, v_b) \in E'\cup {\hat{E}}'$ ($a,b \in \{h, t\}$). By removing edge *e* and leaf node $v_b$, the subgraph $G'_n=(E'-\{e\}, {\hat{E}}'-\{e\}, V'-\{v_b\})$ is also acyclic and contains *n* edges. According to the assumption, $G'_n$ is a compatible structure and there is an arrangement of the segments in which all edges in $E'\cup {\hat{e}}'-\{e\}$ is concordant. Because no other edge in $E'\cup {\hat{E}}'$ except *e* connects to $v_b$, it is always possible to place segment *v* back to the arrangement such that *e* is concordant. Specifically, one of the four placing options will satisfy edge *e*: the beginning of the arrangement with orientation 1, the beginning with orientation 0, the end with orientation 1 and the end with orientation 0. Therefore, $G'_{n+1}$ is a compatible structure.

By induction, acyclic subgraph $G'_N$ of GSG with any $|E'|$ is a compatible structure. $\square$

### **Theorem 5**

*A simple cycle* $C = (E', {\hat{E}}', V')$*is a compatible structure if and only if there are exactly two vertices,* $v_j$*and* $v_i$*such that* $deg_{E'}(v_i) = deg_{E'}(v_j) = 2$*and* $v_i$*and* $v_j$*belongs to different segments.*

### *Proof*

We prove sufficiency and necessity separately in Lemma 1 and Lemma 2. $\square$

### **Lemma 1**

*IfCis a compatible structure, there are exactly two vertices,* $v_i,~v_j$*that belong to different segments, such that* $deg_{E'}(v_i) = deg_{E'}(v_j) = 2$

### *Proof*

We discuss compatibility in two cases:

**Case (1):***All edges are concordant inC*. Sort the vertices by genomic locations in ascending order and label the first vertex $v_1$ and the last $v_n$, assuming $|V'|=n$. Similarly, sort the set of segments $S'$ in *C* by the values of their permutation function $\pi$ and label the first segment $s^1$ and the last $s^m$, assuming $|S'|= m$. Since concordant connections can only be tail-head connections (e.g. Figure 1 b,c), $v_1 = s^1_t$ and $v_n = s^m_h$. Since *C* is a simple cycle, all vertices $v\in V'$ have $deg(v) = 2$. Because $v_1$ and $v_n$ are the first and last vertices in this arrangement, the edges incident to $v_1$ or $v_n$ must be in $E'$. It follows that the two edges incident to $v_1$ connects to $s^2_h$ and $s^m_h$. Similarly, edges incident to $v_n$ connects to $s^1_t$ and $s^{n-1}_t$. Therefore, we have $deg_{E'}(v_1) = deg_{E'}(v_n) = 2$. Any other vertex $v_i$ ($1<i<n$) is connected by one $e\in E'$ and one ${\hat{e}}\in \hat{E'}$ and thus has $deg_{E'}(v_i) = 1$.

**Case (2):***Some edges are discordant inC*. If discordant edges exist in cycle *C*, according to the definition of compatible structure, segments in *C* can be arranged such that all edges are concordant. This reduces to case (1). $\square$

### **Lemma 2**

*If there are exactly two vertices in*$V'$*that belong to different segments,*$v_i$*and*$v_j$, *such that*$deg_{E'}(v_i) = deg_{E'}(v_j) = 2$, *thenCis a compatible structure.*

### *Proof*

Let $v_i$ and $v_j$ be the one of the end points of segments $s^i$ and $s^j (i\ne j)$ , respectively. We can arrange $s^i$ and $s^j$ such that $\pi (s^i) = \min _{s\in S'} \pi (s)$, $\pi (s^j) = \max _{s\in S'} \pi (s)$ and that $v_i = s^i_t$, $v_j = s^j_h$. Rename $v_i$ to $v_1$ and $v_j$ to $v_n$. Since *C* is a simple cycle, we can find two simple paths, $P_1$ and $P_2$, between $v_1$ and $v_n$ and there is no edge between $P_1$ and $P_2$. Let $P_1'$ and $P_2'$ denote $P_1$ and $P_2$ that exclude $v_1$ and $v_n$ and the edges incident to $v_1$ and $v_n$. Since $P_1'$ and $P_2'$ as acyclic subgraphs of GSG, according to Theorem 4, $P_1'$ and $P_2'$ are compatible structures and therefore segments in $P_1'$ and $P_2'$ can be arranged so that all edges are concordant. Denote the first and last vertices in the arranged $P_1'$ as $v_2$ and $v_3$, and the first and last vertices in the arranged $P_2'$ as $v_4$ and $v_5$. Because all the edges are concordant in $P_1'$, $v_2$ and $v_3$ are the head and tail of the first and last segments in $P_1'$. Because only $v_1$ and $v_n$ have $deg_{E'}=2$ in *C*, $v_2$ must be connected to $v_1$ or $v_n$ and $v_3$ must be connected to $v_n$ or $v_1$. A similar argument applies to $v_4$ and $v_5$. To ensure concordance of edges connected to $v_1$ and $v_n$, if $v_n$ is connected to $v_2$ and $v_1$ is connected to $v_3$, we flip all the segments in $P_1'$. The similar operation is applied to $v_4$, $v_5$ and $P_2'$. Now we have a compatible structure. $\square$

### **Corollary 3**

*A necessary condition for a subgraph* $(E', {\hat{E}}', V')$*to be a conflict structure is that it contains cycles. A sufficient condition for a subgraph* $(E', {\hat{E}}', V')$*to be a conflict structure is that it contains a simple cycle which is not a compatible structure.*

The corollary is a direct derivation from Theorem 4 and Theorem 5 when considering general graph structures.

In practice, we determine if a discordant edge, $e=(u,v)$, is involved in a conflict structure by enumerating all simple paths using a modified depth-first search implemented in Networkx [19, 20] between *u* and *v* omitting edge *e*. We add *e* to each path and form a simple cycle. If the simple cycle satisfies Corollary 3, we stop path enumeration and label the *e* as discordant edge involved in conflict structure. If the running time of path enumeration exceeds 0.5 seconds, we shuffle the order of DFS and repeat the enumeration. If path enumeration for *e* exceeds 1000 reruns, we label *e* as undecided.

## Results

To produce an efficient, practical algorithm for TSV detection in diploid organisms, we use the following approach, which we denote as D-SQUID: Run the ILP under the diploid assumption by setting $k=2$ on every connected component of GSG separately. If the ILP finishes or the running time of the ILP exceeds one hour, output the current arrangements.

### D-SQUID identifies more TSVs in TCGA samples than SQUID

**Fig. 2:**
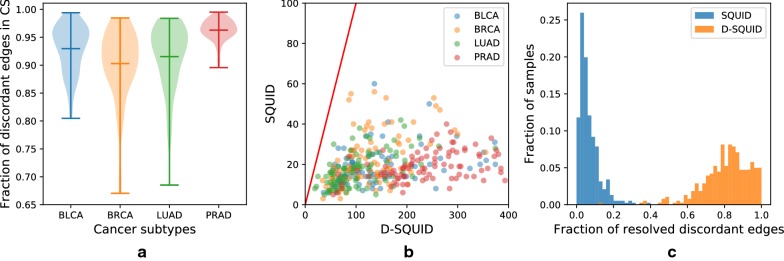
Performance of D-SQUID and SQUID on TCGA samples. **a** The distribution of fractions of discordant edges that are involved in each identified conflict structure (CS) in four cancer subtypes. Minima, maxima and means of the distributions are marked by horizontal bars. **b** Number of TSVs identified by SQUID versus D-SQUID. The red line denotes equality between the number of TSVs between SQUID and D-SQUID. **c** Histogram of fractions of resolved discordant edges by SQUID and D-SQUID

We calculate the fraction of discordant edges involved in conflict structures (Fig. 2a) in 381 TCGA samples from four types of cancers: bladder urothelial carcinoma (BLCA), breast invasive carcinoma (BRCA), lung adenocarcinoma (LUAD) and prostate adenocarcinoma (PRAD). Among all samples, we found less than 0.5% undecided edges out of all discordant edges. The distribution of fraction of discordant edges within conflict structures are different among cancer types. The more discordant edges are involved in conflict structures, the more heterogeneous the sample is. Among four cancer types, PRAD samples exhibit the highest extent of heterogeneity and BRCA samples exhibit the lowest. On average, more than 90% of discordant edges are within conflict structures in all samples across four cancer types. This suggests that TCGA samples are usually heterogeneous and may be partially explained by the fact that TCGA samples are usually a mixture of tumor cells and normal cells [21].

We compare the number of TSVs found by D-SQUID and SQUID (Fig. 2b). In all of our results, all of the TSVs found by SQUID belong to a subset of TSVs found by D-SQUID. D-SQUID identifies many more TSVs than SQUID on all four types of cancers.

A discordant edge is termed resolved if it is made concordant in one of the arrangements. Among all discordant edges in all samples, D-SQUID is able to resolve most of them (Fig. 2c), while SQUID is only able to resolve fewer than 50% of them. The results demonstrate that D-SQUID is more capable of resolving conflict structures in heterogeneous contexts, such as cancer samples, than SQUID.

### D-SQUID identifies more true TSV events than SQUID in cancer cell lines

**Fig. 3:**
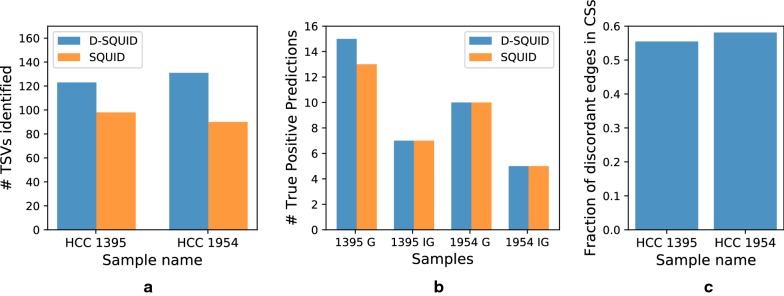
Performance of D-SQUID and SQUID on breast cancer cell lines with experimentally verified SV. **a** Total TSVs found. In both cell line samples, D-SQUID discovered more TSVs than SQUID. **b** Number of known fusion-gene and non-fusion-gene events recovered by D-SQUID and SQUID. G denotes TSVs that affect gene regions. IG denotes TSVs that affect intergenic regions. **c** Fraction of discordant edges in conflict structures

We compare the ability of D-SQUID and SQUID to detect fusion-gene and non-fusion-gene events on previously studied breast cancer cell lines HCC1395 and HCC1954 [22]. The annotation of validated TSVs is taken from Ma et al. [9]. In both cell lines, D-SQUID discovers more TSVs than SQUID. In HCC1954, D-SQUID identifies the same number of known TSVs including fusions of gene (G) regions and intergenic (IG) regions compared with SQUID. In HCC1395, D-SQUID identifies 2 more true TSV events that are fusions of genic regions. We tally the fraction of discordant edges in conflict structures (Fig. 3c) and find similar fractions between HCC1395 and HCC1954, which indicates that the extent of heterogeneity in two samples are similar. Compared to Fig. 2a, the fraction in HCC samples is much lower than that in TCGA samples. This matches the fact that two HCC samples contain the same cell type and are both cell line samples, which are known to be less heterogeneous than TCGA samples.

### D-SQUID predicts TSVs in biologically significant genes in cancer cell lines

**Fig. 4:**
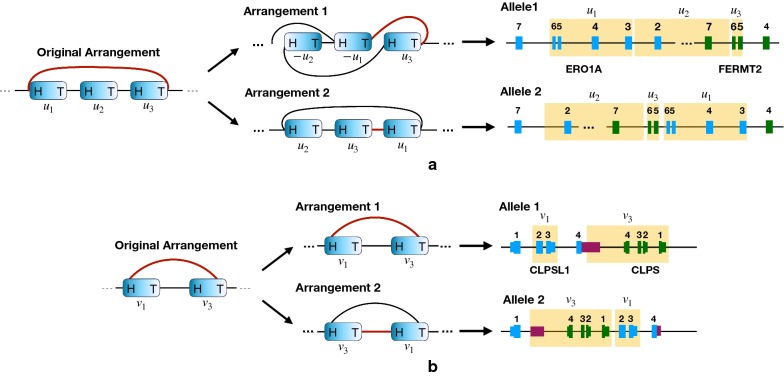
Examples on which D-SQUID predicts a validated (**a**) and an unvalidated (**b**) TSV event that impacts biologically significant genes. The blue blocks represent segments in the GSG. The red edges mark the discordant edges in the original arrangement. Blue and green blocks mark exons of different genes and dark purple blocks mark UTRs in (**b**). Regions highlighted in yellow in the gene models mark the corresponding segments in GSG

Figure 4 gives two examples of TSVs predicted by D-SQUID but not by SQUID. Such TSVs are involved in conflict structures and can only be resolved by separating discordant edges into different arrangements.

An example of a validated TSV is shown in Fig. 4a. The head-tail connection between segment $u^1$ and $u^3$ conflicts with the tail-head connections between segments $u^1$ and $u^2$ and segments $u^2$ and $u^3$. Such a conflict structure is resolved by separating edge $(u^1_h, u^3_t)$ into the second arrangement. Notice that since no discordant edges are made concordant in the first arrangement, no new TSVs are predicted. Therefore, the corresponding gene model for the first arrangement is the same as that of the original arrangement. The affected regions are exons of ERO1A and FERMT2 genes. As predicted by D-SQUID, this TSV involves an insertion of the sixth and the seventh exons of FERMT2 between the sixth and seventh exons of ERO1A.

Among the unvalidated TSVs predicted by D-SQUID, some of them affect genes that are associated with breast cancer. The TSV shown in Fig. 4b involves an insertion of the 3’ untranslated region (UTR) of CLPSL1 and the entire CLPS gene between the first and second exons of CLPSL1. It has been reported that CLPSL1 is associated with a prognostic factor of breast cancer [23].

A full list of affected regions in HCC samples can be found in Additional file 1.

### Evaluation of approximation algorithms

We evaluate the approximation algorithms for diploid MCAP ($k=2$) using two different subroutines described in previous sections. In this subsection, *A*1 refers to using Algorithm 1 with worst case runtime *O*(|*V*||*E*|) as a subroutine and *A*2 refers to using Algorithm 2 with worst case runtime $O(|V|^2|E|)$ as a subroutine. Both *A*1 and *A*2 solve SCAP by greedily inserting segments into the best position in the current ordering. While *A*1 only looks at the beginning and ending of the ordering, *A*2 looks at all the positions.

**Fig. 5:**
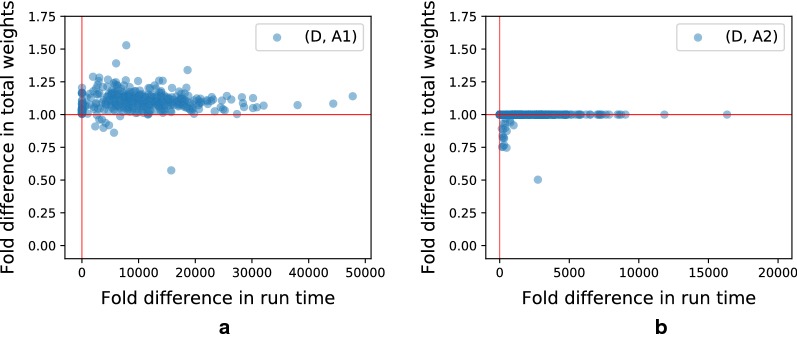
Fold differences (ILP/approx) in run time and total weights of concordant edges resolved by D-SQUID, *A*1 and *A*2 on TCGA samples. Horizontal and vertical red lines mark 1.0 on both axes. **a** Shows fold differences between D-SQUID and *A*1. **b** Shows fold differences between D-SQUID and *A*2

In order to compare the performance of approximations to the exact algorithm using ILP, we run D-SQUID, *A*1 and *A*2 on TCGA samples. The algorithms are evaluated on runtime and total weight of concordant edges in the rearranged genomes. “Fold difference” on the axes of Fig. 5 refers to the ratio of the axis values of D-SQUID over that of *A*1 or *A*2. Both *A*1 and *A*2 output results in a much shorter period of time than D-SQUID. *A*2 achieves better approximation than *A*1, demonstrated by closer-to-one ratio of total concordant edge weight, at a cost of longer run time.

The run time of D-SQUID ILP exceeds 1 h on 4.5% of all connected components in all TCGA samples. D-SQUID outputs sub-optimal arrangements in such cases. As a result, approximation algorithms, especially *A*2, appear to resolve more high-weight discordant edges than D-SQUID in some of the samples in Fig. 5, which is demonstrated by data points that fall below 1 on the y axes. *A*1 resolves more high-weight edges in 10 samples and *A*2 resolves more high-weight edges in 54 samples than D-SQUID.

## Conclusions

We present approaches to identify TSVs in heterogeneous samples via the multiple compatible arrangements problem (MCAP). We characterize sample heterogeneity in terms of the fraction of discordant edges involved in conflict structures. In the majority of TCGA samples, the fractions of discordant edges in conflict structures are high compared to HCC samples, which indicates that TCGA samples are more heterogeneous than HCC samples. This matches the fact that bulk tumor samples often contain more heterogeneous genomes than cancer cell lines, which suggests that fraction of conflicting discordant edges is a valid measure of sample heterogeneity.

We show that obtaining exact solutions to MCAP is NP-complete. We derive an integer linear programming (ILP) formulation to solve MCAP exactly. We provide a $\frac{3}{16}$-approximation algorithm for MCAP when the number of arrangements is two ($k=2$), which runs in time *O*(|*V*||*E*|). It approximates the exact solutions well in TCGA samples.

MCAP addresses this heterogeneity. In 381 TCGA samples, D-SQUID is able to resolve more conflicting discordant edges than SQUID. Since D-SQUID solves MCAP by separating conflicting TSVs onto two alleles, D-SQUID’s power to find TSVs generally increases as the extent of heterogeneity increases. In HCC cell lines, D-SQUID achieves better performance than SQUID. Aside from validated TSV events, D-SQUID discovers unvalidated fusion-gene events that impact genes associated with cancer, which requires further investigation.

Several open problems remain. MCAP relies on the number of arrangements (*k*) to make predictions. It is not trivial to determine the optimal *k* for any sample. In addition, although MCAP is solved by separating TSVs onto different alleles, there are typically many equivalent phasings. Developing techniques for handling these alternative phasings is an interesting direction for future work. Analyzing the effect of TSVs, especially non-fusion-gene ones, on their impact on cellular functions and diseases is another direction of future work.

Another potential future direction to improve the accuracy of TSV prediction is to incorporate the distance between breakpoints and read pairs into the optimization formulation. A long distance between read pairs mapped to the reference genome indicates a potential TSV event induced by deletion events. Ignoring such long distances leads to false negatives. On the other hand, long distances between breakpoints of a fusion-gene TSV in the rearranged genome can potentially indicate false positive predictions. We show that thresholding distances during pre- and post-processing steps of D-SQUID is helpful in reducing false negatives, but not as effective in reducing false positives partially due to the lack of distance consideration in the current problem formulation (Additional files 1, 2). Investigating and evaluating potential ways to incorporate the distance information, such as adding a distance threshold to the edge concordance definition or adding distance penalties into the ILP, is a future direction for improvement.

## Supplementary information

**Additional file 1. Annotated break points predicted by D-SQUID in HCC cell line samples.** Break points predicted by D-SQUID and the gene regions they react in HCC1395 and HCC1954 samples.

**Additional file 2. Supplementary document on distance considerations.** An extended discussion on the false positives and false negatives that may be due to lack of consideration about various distances in the genome.

## Data Availability

HCC1395 and HCC1954 sequencing data analyzed during the current study are available in the SRA repository. The accession numbers are HCC1395 RNA-seq: SRR2532336 [25] HCC1954 RNA-seq: SRR2532344 [26] and SRR925710 [27] TCGA WGS and RNA-seq data are available through application to dbGaP [28].

## Abbreviations

BLCA: Bladder urothelial carcinoma
CS: Conflict structure
BRCA: Breast invasive carcinoma
GSG: Genome Segment Graph
ILP: Integer linear programming
LUAD: Lung adenocarcinoma
MCAP: Multiple Compatible Arrangements Problem
PRAD: Prostate adenocarcinoma
SCAP: Single Compatible Arrangement Problem
SRA: Sequencing Read Archive
TCGA: The Cancer Genome Atlas
TSV: Transcriptomic structural variant
