# Supplementary material for: Detecting transcriptomic structural variants in heterogeneous contexts via the Multiple Compatible Arrangements Problem
Source: Algorithms Mol Biol. 2020 May 15;15:9. doi: 10.1186/s13015-020-00170-5 (PMC7227063; doi:10.1186/s13015-020-00170-5)
Supplement: Supplementary file 2 — Additional file 2. Supplementary document on distance considerations. An extended discussion on the false positives and false negatives that may be due to lack of consideration about various distances in the genome. [file 13015_2020_170_MOESM2_ESM.pdf]

SUPPLEMENTARY DOCUMENT

Detecting Transcriptomic Structural Variants in Heterogeneous Contexts via the Multiple Compatible Arrangements Problem

Yutong Qiu†, Cong Ma†, Han Xie and Carl Kingsford*

*Correspondence:
carlk@cs.cmu.edu
Computational Biology Department, Carnegie Mellon University, 5000 Forbes Ave, 15213 Pittsburgh, PA, USA
Full list of author information is available at the end of the article
†Equal contributor

Distance Considerations

False negatives

False negatives are validated TSVs in HCC samples that are not predicted by D-SQUID. In the current Genome Segment Graph (GSG) construction algorithm, a read pair is considered concordant regardless of the distance between the pair. Therefore, read pairs that are far apart may end up in one genome segment in GSG and thus ignored by the downstream arrangement algorithm. Although we predict the edges that connect far apart segments in the original genome as TSVs as a post-processing step, it is possible that some deletion events are ignored.

We investigate the false negatives that may correspond to those ignored deletion events. Among 128 false negatives in HCC1395, for 67 of them, both of their breakpoints locate within one segment. For the remaining 61 false negatives, each pair of breakpoints locate in two segments that are not connected to each other, which means that the breakpoints are not supported by any read. Among 46 false negatives in HCC1954, for 21 of them, both of their breakpoints locate within one segment and 25 of them are not supported by any edge. In both samples, none of the false negatives are represented by concordant or discordant edges (Table 1). This shows that the additional step that outputs concordant edges that connect far apart segments is effective in reducing false negatives.

Among the false negatives whose breakpoints locate within one segment, 3 of them are supported by reads in HCC1395 and 4 of them are supported by reads in HCC1954 (Table 2). In HCC1395, the distance between those breakpoints are

greater than the average gene length, which may correspond to deletion events. In HCC1954, some of the distances are small, which may correspond to other TSV events.

The results show that most of the false negatives lack read support. Still, adding read-pair and segment distance as a concordancy consideration may reduce false negatives such as the TSVs in HCC1395 listed in Table 2.

Table 1 Different types of false negatives in HCC1395 and HCC1954 samples.

Category of False Negative TSVs	HCC1395	HCC1954
**Within one node**	67	21
**Not supported by any edge**	61	25
**Concordant**	0	0
**Discordant**	0	0

Table 2 Within-one-node false negative TSVs that are supported by more than one read pair in HCC samples.

Sample	Chr 1	Position 1	Chr 2	Position 2	Distance between breakpoints
**HCC1395**	20	3843059	20	3888573	45513
	11	36120010	11	36422547	302536
	16	85391248	16	85667520	276271
**HCC1954**	17	37647567	17	37648275	707
	17	37815692	17	37816068	375
	6	35754695	6	35766710	12014
	5	6730963	5	6730963	0

False positives

False positives are the predicted TSVs in D-SQUID that are not validated by the ground truth. We investigate whether the distance between the two segments in a TSV can be used to distinguish between true positive (TP) and false positive (FP) predictions in D-SQUID.

Two types of distances are considered: the number of segments between each TSV-connected segments (node distance), and the number of basepairs between each TSV-connected segments (basepair distance) in the output arrangement. D-SQUID outputs two arrangements of segments that correspond to the diploid assumption. When a discordant edge is made concordant in one of the arrangements, that arrangement where the edge is concordant is used in counting segments and basepairs for the distance calculation. When a discordant edge is made concordant in both arrangements, we use the arrangement where the basepair distance is the minimum to calculate the distances. We choose a smaller basepair distance because the actual

distance in the true rearranged genome cannot be too large. A minimum basepair distance within the two output arrangements is an upper bound of the minimum across all co-optimal solutions.

The predictions with the largest node distances and the largest basepair distances tend to be FP predictions (Supplementary Figure S1). This indicates that directly applying a node distance and a basepair distance threshold on the rearranged genome is able to reduce the FP predictions.

Nevertheless, the number of reduced FP is limited, since the distributions of the distances of TP and FP predictions both have a large mass at small distances (Supplementary Figure S1B, C) and the tail of FP distance distribution is very small. In addition, without investigating more validated datasets, a widely applicable distance threshold cannot be determined. This limitation is partially because D-SQUID and SQUID may not choose the arrangements with the minimum distances among all co-optimal arrangements. But if a distance penalty is included in D-SQUID or SQUID objective for selecting arrangements from co-optima, TP and FP may be better distinguished.

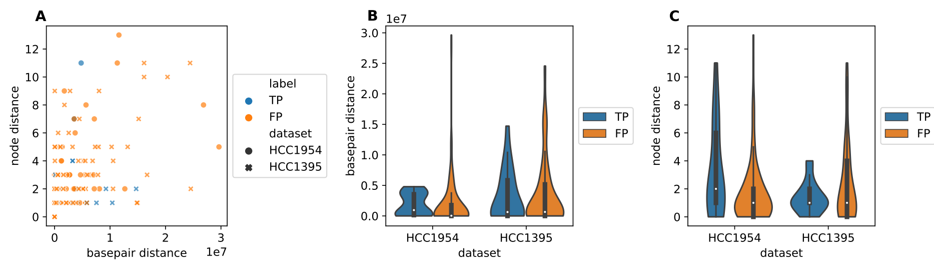

Figure S1 Distances of true positive (TP) and false positive (FP) predictions using number of nodes and number of basepairs. (A) Scatter plot between basepair distance (x axis) and node distance (y axis). Each point is a predicted TSV by D-SQUID. Shape of the points represents the dataset and color indicates whether the prediction is TP or FP. (B) Violin plot of the distribution of basepair distance for TP and FP predictions in both HCC datasets. (C) Violin plot of the distribution of node distance for TP and FP predictions in both HCC datasets. bp distance here refers to basepair distance between connected segments.
